# Growth, tolerance and safety outcomes with use of an extensively hydrolyzed casein-based formula in infants with cow’s milk protein allergy

**DOI:** 10.3389/fped.2023.1230905

**Published:** 2023-07-31

**Authors:** Aydan Kansu, Nafiye Urganci, Aysegul Bukulmez, Gunsel Kutluk, Didem Gulcu Taskin, Lutfiye Sahin Keskin, Mahir Igde, Lutfi Molon, Yasar Dogan, Bulent Enis Sekerel, Mutlu Yuksek, Ilknur Bostanci, Nelgin Gerenli, Esra Polat, Buket Dalgic, Hasret Ayyildiz, Merve Usta, Ahmet Basturk, Ozlem Yuce Kirmemis, Ceyda Tuna Kirsaclioglu, Hacer Fulya Gulerman, Aysugul Alptekin Sarioglu, Simge Erdogan

**Affiliations:** ^1^ Department of Pediatric Gastroenterology, School of Medicine, Ankara University, Ankara, Turkey; ^2^ Clinic of Pediatric Gastroenterology, Istanbul Sisli Hamidiye Etfal Training and Research Hospital, Istanbul, Turkey; ^3^ Department of Pediatrics, Afyonkarahisar Health Sciences University, Afyonkarahisar, Turkey; ^4^ Clinic of Pediatric Gastroenterology, Istanbul Kanuni Sultan Suleyman Training and Research Hospital, Istanbul, Turkey; ^5^ Clinic of Pediatric Gastroenterology, Adana City Training and Research Hospital, Adana, Turkey; ^6^ Department of Pediatric Allergy and Immunology, Faculty of Medicine, Istinye University, Istanbul, Turkey; ^7^ Department of Pediatric Gastroenterology, Faculty of Medicine, Firat University, Elazig, Turkey; ^8^ Department of Pediatric Allergy, Faculty of Medicine, Hacettepe University, Ankara, Turkey; ^9^ Department of Pediatric Immunology and Allergy, Faculty of Medicine, Zonguldak Bulent Ecevit University, Zonguldak, Turkey; ^10^ Clinic of Pediatric Immunology and Allergy, Health Sciences University Dr. Sami Ulus Gynecology, Obstetrics and Child Health and Diseases Training and Research Hospital, Ankara, Turkey; ^11^ Clinic of Pediatric Gastroenterology, Istanbul Umraniye Training and Research Hospital, Istanbul, Turkey; ^12^ Department of Pediatric Gastroenterology, Faculty of Medicine, Gazi University, Ankara, Turkey; ^13^ Clinic of Pediatric Gastroenterology, Istanbul Bakirkoy Dr. Sadi Konuk Training and Research Hospital, Istanbul, Turkey; ^14^ Department of Pediatric Gastroenterology, Faculty of Medicine, Gaziantep University, Gaziantep, Turkey; ^15^ Clinic of Pediatric Gastroenterology, Samsun Training and Research Hospital, Samsun, Turkey; ^16^ Department of Pediatric Gastroenterology, Faculty of Medicine, Kirikkale University, Kirikkale, Turkey; ^17^ Abbott Nutrition, Abbott Laboratories, Istanbul, Turkey

**Keywords:** cow’s milk protein allergy, extensively hydrolyzed casein-based formula, growth indices, gastrointestinal intolerance, stool patterns, parental satisfaction

## Abstract

**Objective:**

To evaluate growth, tolerance and safety outcomes with use of an extensively hydrolyzed casein-based formula (eHCF) in infants with cow’s milk protein allergy (CMPA).

**Methods:**

A total of 226 infants (mean ± SD age: 106.5 ± 39.5 days, 52.7% were girls) with CMPA who received eHCF comprising at least half of the daily dietary intake were included. Data on anthropometrics [weight for age (WFA), length for age (LFA) and weight for length (WFL) *z*-scores] were recorded at baseline (visit 1), while data on infant feeding and stool records, anthropometrics and Infant Feeding and Stool Patterns and Formula Satisfaction Questionnaires were recorded at visit 2 (on Days 15 ± 5) and visit 3 (on Days 30 ± 5).

**Results:**

From baseline to visit 2 and visit 3, WFA *z*-scores (from −0.60 ± 1.13 to −0.54 ± 1.09 at visit 2, and to −0.44 ± 1.05 at visit 3, *p* < 0.001) and WFL *z*-scores (from −0.80 ± 1.30 to −0.71 ± 1.22 at visit 2, and to −0.64 ± 1.13 at visit 3, *p* = 0.002) were significantly increased. At least half of infants never experienced irritability or feeding refusal (55.7%) and spit-up after feeding (50.2%). The majority of mothers were satisfied with the study formula (93.2%), and wished to continue using it (92.2%).

**Conclusions:**

In conclusion, eHCF was well-accepted and tolerated by an intended use population of infants  ≤ 6 months of age with CMPA and enabled adequate volume consumption and improved growth indices within 30 days of utilization alongside a favorable gastrointestinal tolerance and a high level of parental satisfaction.

## Introduction

Cow’s milk protein allergy (CMPA), a reproducible clinically abnormal reaction to cow’s milk protein (CMP), is a common food allergy in the pediatric age (1, 2). CMPA usually presents within the first six months of life with gastrointestinal, cutaneous, and/or respiratory manifestations or anaphylaxis (1–3).

The principles of CMPA treatment include avoidance of CMP in the mothers of breastfed infants and the provision of substitute formulae adapted to CMPA dietary management in terms of allergic and nutritional safety in formula-fed infants (2–7). Extensively-hydrolyzed casein-based infant formulas (eHCF) with demonstrated hypoallergenicity and favorable tolerance are therefore recommended by guidelines as a first line treatment for infants and children with confirmed CMPA (4–8).

The safety and efficacy of extensively hydrolyzed formula (eHFs) compared to other treatment formulas has been emphasized particularly in terms of the decreased incidence of CMPA-related symptoms and better tolerance (9, 10). However, CMPA may also result in growth retardation due to several factors including the impact of the allergic manifestations on nutritional status, increased metabolic needs, disrupted sleep patterns, gut inflammation causing impaired nutrient bioavailability, and a restricted diet (5, 11–15).

In this regard, eHFs should also be investigated for their growth adequacy in children with CMPA, since children with CMPA are considered to be at increased risk of poor growth (5, 11, 14, 15). Indeed, the assessment of growth and development with eHFs is considered to be a critical safety parameter, given the likelihood of slower weight and length growth with eHFs compared to the amino acid-based formula in the setting of CMPA (16–18).

However, while the efficacy and tolerability of eHCF in the management of infants and children with CMPA have been demonstrated, its suitability for growth in infants with CMPA has not been sufficiently investigated (11, 17, 19–21).

This prospective observational multicenter study aimed to evaluate the utility of an eHCF in an intended-use population of infants with CMPA in terms of the growth indices, gastrointestinal intolerance and safety as well as the parent’s assessment of infant feeding and stool patterns and formula satisfaction.

## Material and methods

### Study population

A total of 226 infants (mean ± SD age: 106.5 ± 39.5 days, 52.7% were girls) with CMPA who received eHCF comprising at least half of the daily dietary intake were included in this non-randomized, single-arm, multicenter clinical trial conducted at 35 pediatric gastroenterology and allergy centers across Turkey. The study sample was composed of infants (aged 0–180 days) with newly diagnosed CMPA where an eHCF was deemed appropriate by the healthcare professional for at least 50% of their feeding with breastfeeding comprising the other half, as confirmed by parents’ records on daily frequency of feeding. None of the infants were on the complementary feeding. Although 320 infants were initially enrolled, the final study population was composed of 226 infants with the exclusion of 94 infants due to protocol violation and/or loss to follow up (*n* = 72), discontinuation of the formula due to dissatisfaction (*n* = 13), consent withdrawal (*n* = 3), adverse event development (*n* = 1) and other reasons (*n* = 5).

Written informed consent was obtained from the parent/legal guardian of each subject following a detailed explanation of the objectives and protocol of the study, which was conducted in accordance with the ethical principles stated in the “Declaration of Helsinki” and approved by the Ankara University Faculty of Medicine Clinical Research Ethics Committee (Date of Approval: 24/07/2017; Reference number/Protocol No: 12-703-17).

### Maternal diet

Mothers followed a CMP-free diet by avoiding all milk and milk products like cheese, yogurt and butter as well as the foods that may contain traces of milk, from the maternal diet and received calcium supplements in accordance with the qualified dietary counseling.

### Assessments

Data on demographics, clinical history and anthropometrics [*z*-scores for weight for age (WFA), length for age (LFA) and weight for length (WFL)] were recorded at baseline (visit 1). The infant feeding, spit-up (mild vomiting or regurgitation of milk, formula and saliva in small amounts) or vomiting (throwing up of the stomach contents with force and muscle contractions) and stool records, anthropometrics and study questionnaires (Infant Feeding and Stool Patterns and Formula Satisfaction Questionnaires) as well as safety (adverse events) were evaluated at visit 2 (on days 15 ± 5) and visit 3 (on days 30 ± 5). Outcome measures included infant growth, gastrointestinal tolerance, safety and parental satisfaction with eHCF in the overall study population, as well as in the subgroups of exclusively formula-fed and mixed-fed infants (Table 1).

**Table 1:** Study flowchart.

Assessments	Visit 1 (day 1)^a^	Day 5–7^b^	Visit 2 (day 15)^c^	Visit 3 (day 30)^c^
Enrollment	**X**			
Demographic data	**X**			
Clinical history	**X**			
Anthropometric measurements	**X**		**X**	**X**
Intake records, Stool records^d^			**X**	**X**
Telephone follow-up		**X**		
Study questionnaires	**X**		**X**	**X**
Interval history			**X**	**X**
Adverse events	**X**		**X**	**X**

^a^ Date of birth is Day zero of life (enrollment 0–180 days of age where Days of age = enrollment date minus date of birth).

^b^ Telephone follow-up window.

^c^ Visit window ± 5 days.

^d^ Intake and stool records were maintained by parent(s) beginning with the first study feeding after Visit 1 and were collected each day afterwards.

Infant growth was assessed based on the maintenance of anthropometric *z*-scores (using WHO reference data) during the study (22). Formula intake and stool records were requested to be kept by the mother beginning with the first study formula feeding after Visit 1 and were collected daily thereafter. Gastrointestinal tolerance and compliance of the study product were also assessed along with parental satisfaction via responses to the Infant Feeding and Stool Patterns and Formula Satisfaction Questionnaires.

### Growth indices

Anthropometric measurements included body weight (kg) and length (cm) along with the calculation of mean *z*-scores for WFA, LFA and WFL. At each study visit, body weight was measured using the same digital baby weight scale (10 g precision) and height measurement was performed using a 1-m length measuring tape (0.1 cm precision), while the use of an infant stadiometer was at the discretion of the physician.

### Statistical analysis

Statistical analysis was made using IBM SPSS Statistics for Windows, Version 23.0 (IBM Corp., Armonk, NY). Chi-square (*χ*^2^) test was used for the comparison of categorical data, while numerical data were analyzed using the Friedman test. Change over time for continuous data was evaluated by Wilcoxon signed rank test or paired *t*-test. Change over time for categorical data was analyzed using McNemar’s test or Bowker’s test. Data were expressed as mean ± SD (standard deviation), median (minimum-maximum) and percent (%) where appropriate. A *p*-value <0.05 was considered statistically significant.

## Results

### Baseline characteristics

Mean ± SD age of infants was 106.5 ± 39.5 days, and 52.7% were girls.

At the time of enrolment, 78.3% of infants were fed by breast milk (with avoidance of CMP in the mothers) + eHCF (mixed-fed group), while 21.7% were exclusively fed by eHCF (exclusively formula-fed group) (Table 2).

**Table 2:** Demographic and clinical characteristics (*n* = 226).

		Overall	Exclusively formula-fed (*n* = 49)	Mixed-fed (*n* = 177)
Demographic characteristics				
Age (day)	mean ± SD	106.5 ± 39.5	100.7 ± 41.9	108.1 ± 38.8
	median (min-max)	106.5 (20–179)	100 (24–178)	114 (20–179)
Gender, *n* (%)				
Male		107 (47.3)	22 (44.9)	85 (48.0)
Female		119 (52.7)	27 (55.1)	92 (52.0)
Clinical characteristics, *n* (%)				
Type of feeding at enrolment				
Breastmilk + formula		177 (78.3)	–	177 (78.3)
Formula only		49 (21.7)	49 (21.7)	–
Follow up clinic, *n* (%)				
Pediatric gastroenterology		169 (74.0)	40 (81.6)	129 (72.9)
Pediatric allergy		57 (26.0)	9 (18.4)	48 (27.1)
Clinical manifestations^a^				
GIS		77 (34.1)	16 (32.7)	61 (34.5)
GIS + cutaneous		83 (36.7)	17 (34.7)	66 (37.3)
GIS + cutaneous + respiratory		42 (18.6)	5 (10.2)	37 (20.9)
GIS + respiratory		17 (7.5)	8 (16.3)	9 (5.1)
Cutaneous		6 (2.2)	3 (6.1)	3 (1.7)
Cutaneous + respiratory		1 (0.4)	–	1 (0.6)
Multiple food allergies				
Yes (egg allergy in 15 patients)		20 (8.8)	1 (2.0)	19 (10.7)
No		206 (91.2)	48 (98.0)	158 (89.3)

^a^ GIS: Proctocolitis, vomiting, diarrhea, colic, regurgitation, refusal to feed, constipation; Cutaneous: Urticaria, eczema, persistent diaper rash; Respiratory: Nasal discharge, rhinitis, wheezing, cough.

Overall, most of infants presented with gastrointestinal manifestations either alone (34.1%) or together with cutaneous (36.7%) complaints, regardless of the feeding subgroup (exclusively formula-fed or mixed-fed) (Table 2).

Respiratory complaints were more likely to accompany the gastrointestinal + cutaneous complaints in the mixed-fed group (20.9% vs. 10.2%) and the gastrointestinal complaints (16.3% vs. 5.1%) in the exclusively-fed group (Table 2).

Multiple food allergies (≥2 food groups) were evident in 20 (8.8%) infants overall (egg allergy in most cases), which was more likely in the mixed-fed vs. exclusively formula-fed group (10.7% vs. 2.0%) (Table 2).

### Daily volume intake per each feeding session and spit-up or vomiting after feeding

Daily volume intake per each feeding significantly increased from visit 2 to visit 3 in the overall study population (76.1 ± 28.9 vs. 82.9 ± 30.7 ml/per feeding/per day, *p* < 0.001), and in both exclusively formula-fed (84.3 ± 26.2 vs. 94.3 ± 23.7 ml/per feeding/per day, *p* = 0.001) and mixed-fed (72.6 ± 29.5 vs. 78.2 ± 32.3 ml/per feeding/per day, *p* = 0.002) groups (Table 3).

**Table 3:** Daily volume intake per each feeding session and spit-up or vomiting after feeding.

		Visit 2 (day 15)	Visit 3 (day 30)	*p*-value
Daily volume intake per each feeding session, ml				
Overall	N	121	121	**<0**.**001**^a^
	Mean ± SD	76.1 ± 28.9	82.9 ± 30.7	
Exclusively formula-fed	N	36	36	**0**.**001**^b^
	Mean ± SD	84.3 ± 26.2	94.3 ± 23.7	
Mixed-fed	N	85	85	**0**.**002**^b^
	Mean ± SD	72.6 ± 29.5	78.2 ± 32.3	** **
Spit-up after feeding				
Overall	N	121	121	**<0**.**001**^b^
	Yes	86 (71.1)	61 (50.4)	
	No	35 (28.9)	60 (49.6)	
Exclusively formula-fed	N	36	36	0.125^b^
	Yes	27 (75)	22 (61.1)	
	No	9 (25)	14 (38.9)	
Mixed-fed	N	85	85	**<0**.**001**^b^
	Yes	59 (69.4)	39 (45.9)	
	No	26 (30.6)	46 (54.1)	
Vomiting after feeding				
Overall	N	121	121	**0**.**001**^b^
	Yes	74 (61.2)	54 (44.6)	
	No	47 (38.8)	67 (55.4)	
Exclusively formula-fed	N	36	36	0.109^b^
	Yes	23 (63.9)	17 (47.2)	
	No	13 (36.1)	19 (52.8)	
Mixed-fed	N	85	85	**0**.**009**^b^
	Yes	51 (60)	37 (43.5)	
	No	34 (40)	48 (56.5)	

Values in bold indicate statistical significance (*p* < 0.05).

^a^ Paired samples *t*-test.

^b^ Mcnemar test.

Overall, the frequency of spit-up after feeding (71.1% to 50.4%, *p* < 0.001) and vomiting after feeding (61.2% to 44.6%, *p* = 0.001) were significantly decreased from visit 2 to visit 3, particularly in the mixed-fed group (*p* < 0.001 and *p* = 0.009, respectively) (Table 3).

### Anthropometrics

**Figure 1:**
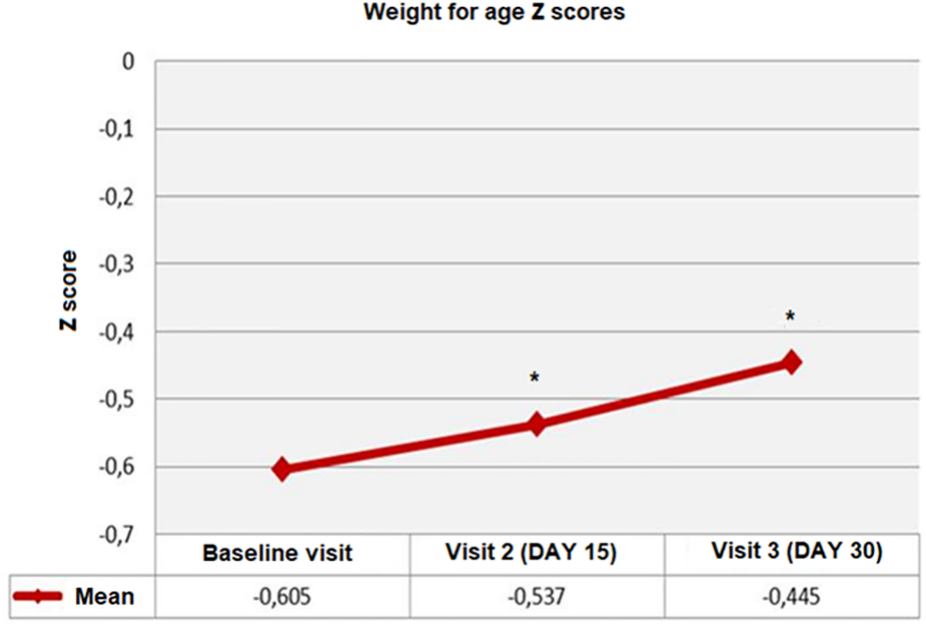
Weight for age (WFA) *Z*-scores from baseline to visit 3, *p < 0.001 compared to baseline visit.

Overall, WFA *z*-scores were significantly increased from baseline to visit 2 and visit 3 (from −0.60 ± 1.13 to −0.54 ± 1.09 at visit 2, and to −0.44 ± 1.05 at visit 3, *p* < 0.001). Mean ± SD WFA *z*-scores (*p* < 0.05) and change from baseline (0.16 ± 0.56 vs. 0.07 ± 0.48, *p* = 0.001) were also significantly higher at visit 3 vs. visit 2 (Table 4, Figure 1).

**Table 4:** Anthropometrics.

Anthropometrics, Mean ± SD		Baseline visit (day 1)	Visit 2 (day 15)	Visit 3 (day 30)	*p*-value
WFA *z*-score					
Overall	N	216	216	216	
	Visit score	−0.60 ± 1.13	−0.54 ± 1.09	−0.44 ± 1.05	**<0**.**001**^a^
	*Change from baseline*		0.07 ± 0.48	0.16 ± 0.56	**0.001** ^b^
Exclusively formula-fed	N	48	48	48	
	Visit score	−0.65 ± 1.25	−0.56 ± 1.08	−0.55 ± 0.96	0.643^a^
	*Change from baseline*		0.09 ± 0.43	0.09 ± 0.59	0.622^b^
Mixed-fed	N	168	168	168	
	Visit score	−0.59 ± 1.09	−0.52 ± 1.09	−0.41 ± 1.07	**<0**.**001**^a^
	*Change from baseline*		0.06 ± 0.49	0.18 ± 0.55	**<0.001** ^b^
LFA *z*-score					
Overall	N	210	210	210	
	Visit score	0.11 ± 1.38	0.18 ± 1.33	0.20 ± 1.24	0.877^a^
	*Change from baseline*		0.07 ± 0.91	0.09 ± 1.13	0.814^b^
Exclusively formula-fed	N	45	45	45	
	Visit score	0.19 ± 1.51	0.37 ± 1.39	0.31 ± 1.13	0.416^a^
	*Change from baseline*		0.18 ± 0.98	0.12 ± 1.32	0.460^b^
Mixed-fed	N	165	165	165	
	Visit score	0.08 ± 1.34	0.12 ± 1.31	0.17 ± 1.27	0.645^a^
	*Change from baseline*		0.04 ± 0.89	0.09 ± 1.08	0.875^b^
WFL *z*-score					
Overall	N	210	210	210	
	Visit score	−0.80 ± 1.30	−0.71 ± 1.22	−0.64 ± 1.13	**0**.**002**^a^
	*Change from baseline*		0.09 ± 0.90	0.16 ± 1.10	**0.026** ^b^
Exclusively formula-fed	N	45	45	45	
	Visit score	−1.04 ± 1.67	−1.00 ± 1.50	−0.89 ± 1.41	0.115^a^
	*Change from baseline*		0.04 ± 1.03	0.15 ± 1.28	0.158^b^
Mixed-fed	N	165	165	165	
	Visit score	−0.74 ± 1.17	−0.63 ± 1.12	−0.57 ± 1.04	**0**.**015**^a^
	*Change from baseline*		0.11 ± 0.87	0.17 ± 1.06	0.081^b^

WFA, weight for age; LFA, length for age; WFL, weight for length.

Values in bold indicate statistical significance (*p* < 0.05).

^a^ Friedman test and post-hoc Wilcoxon test with Bonferroni correction.

^b^ Wilcoxon test.

In the mixed-fed group, mean ± SD WFA *z*-scores were significantly increased from baseline (−0.59 ± 1.09) to visit 2 (−0.52 ± 1.09, *p* < 0.001) and visit 3 (−0.41 ± 1.072, *p* < 0.001), as well as from visit 2 to visit 3 (*p* < 0.05). In the exclusively formula-fed group baseline mean ± SD WFA *z*-scores (−0.65 ± 1.25) were maintained at visit 2 (−0.56 ± 1.08) and visit 3 (−0.55 ± 0.96) (*p* = 0.643) (Table 4).

**Figure 2:**
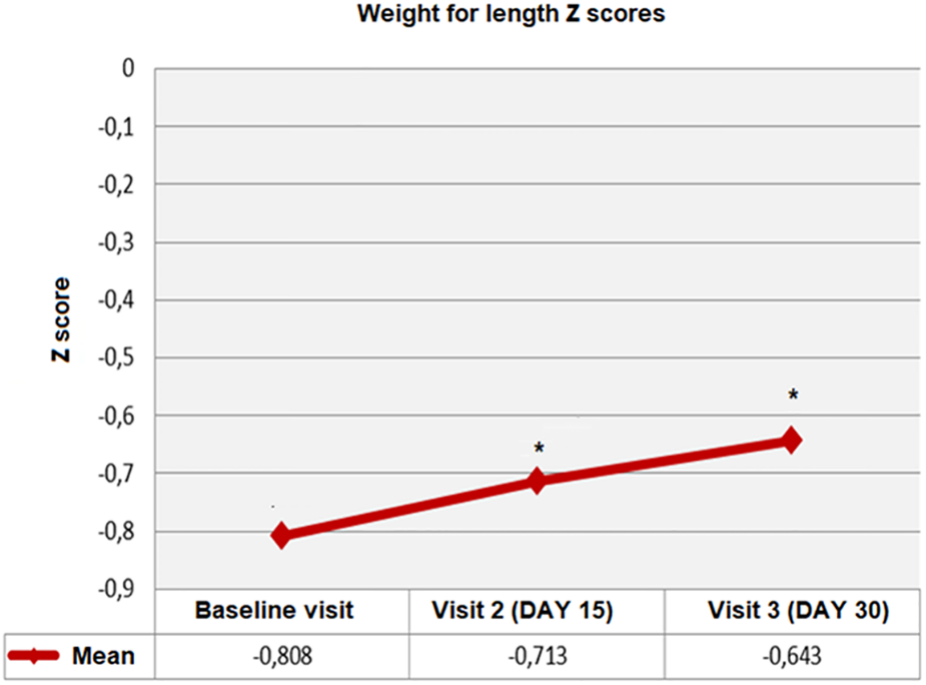
Weight for length (WFL) *Z*-scores from baseline to visit 3, *p < 0.01 compared to baseline visit.

Overall, WFL *z*-scores were significantly increased from baseline to visit 2 and visit 3 (from −0.80 ± 1.30 to −0.71 ± 1.22 at visit 2, and to −0.64 ± 1.13 at visit 3, *p* = 0.002). Mean ± SD change from baseline (0.16 ± 1.10 vs. 0.09 ± 0.90, *p* = 0.026) was also significantly higher at visit 3 vs. visit 2 (Table 4, Figure 2).

In the mixed-fed group, mean ± SD WFL *z*-scores were significantly increased from baseline to follow-up visits (from −0.74 ± 1.173 at baseline to −0.63 ± 1.12 and −0.57 ± 1.04 at visit 2 and 3 respectively; *p* = 0.015). In the exclusively formula-fed group, baseline WFL *z*-scores were maintained at visit 2 and visit 3 (−1.04 ± 1.67 at baseline and −1.00 ± 1.50 and −0.89 ± 1.41 at follow-up visits, respectively; *p* = 0.115) (Table 4).

No significant change was noted in LFA *z*-scores from baseline to follow-up visits overall or in mixed-fed and exclusively formula-fed groups (Table 4).

### Perianal problems, infant feeding and stool patterns

When compared to baseline, the percentage of infants with anal fissure (14.1% vs. 8.2% and 5.4%, respectively, *p* < 0.001) and perianal erythema (37.0% vs. 17.3% and 16.0%, respectively, *p* < 0.001) significantly decreased at visit 2 and visit 3 (Table 5).

**Table 5:** Perianal problems.

Perianal problems		Baseline visit (day 1)	Visit 2 (day 15)	Visit 3 (day 30)	*p*-value
Anal fissure, *n* (%)					
Overall	N	220	220	220	
	Yes	31 (14.1)	18 (8.2)	12 (5.4)	**<0**.**001**
	No	189 (85.9)	202 (91.8)	208 (94.6)	** **
Exclusively formula fed	N	48	48	48	** **
	Yes	3 (6.3)	4 (8.3)	1 (2.1)	0.247
	No	45 (93.7)	44 (91.7)	47 (97.9)	** **
Mixed fed	N	172	172	172	** **
	Yes	28 (16.3)	14 (8.1)	11 (6.4)	**<0**.**001**
	No	144 (83.7)	158 (91.9)	161 (93.6)	** **
Perianal erythema, *n* (%)					
Overall	N	219	219	219	** **
	Yes	81 (37.0)	38 (17.3)	35 (16)	**<0**.**001**
	No	138 (63.0)	181 (82.7)	184 (84)	** **
Exclusively formula fed	N	48	48	48	** **
	Yes	18 (37.5)	7 (14.6)	5 (10.4)	**<0**.**001**
	No	30 (63.5)	41 (85.4)	43 (89.6)	** **
Mixed fed	N	171	171	171	** **
	Yes	63 (36.8)	31 (18.1)	30 (17.5)	**<0**.**001**
	No	108 (63.2)	140 (81.9)	141 (82.5)	** **

Values in bold indicate statistical significance (*p* < 0.05).

Cohran’s *Q*-test and post-hoc McNemar test with Bonferroni correction.

The amelioration of perianal erythema at follow-up visits was significant in both exclusively formula-fed (*p* < 0.001) and mixed-fed (*p* < 0.001) groups, whereas the significant decrease in anal fissure during follow-up visits was only evident in the mixed-fed group (*p* < 0.001) (Table 5).

Visit 3 data on “Infant Feeding and Stool Patterns” related to compliance and gastrointestinal tolerance revealed that at least half of parents perceived that their infants never fussed or resisted the bottle while being fed the formula (55.7%) and never spit-up after feeding (50.2%) along with presence of normal stool consistency always in 54.7%. No significant change was noted between visit 2 and visit 3 in terms of these compliance and gastrointestinal tolerance characteristics in formula-fed and mixed-fed groups (Table 6).

**Table 6:** Infant feeding and stool patterns questionnaire.

Infant feeding and stool patterns^a^	Overall			Exclusively formula fed					Mixed fed				
	Always	Sometimes	Never	*n*	Always	Sometimes	Never	*p*	*n*	Always	Sometimes	Never	*p*
My baby fussed or resisted the bottle while being fed the formula													
Visit 2 (Day 15)	31 (14.9)	72 (34.6)	105 (50.5)	46	5 (10.9)	17 (37)	24 (52.2)	0.478	149	25 (16.8)	51 (34.2)	73 (49)	0.655
Visit 3 (Day 30)	29 (14.3)	61 (30)	113 (55.7)		5 (10.9)	13 (28.3)	28 (60.9)			22 (14.8)	48 (32.2)	79 (53)	
My baby drank formula within a reasonable period of time													
Visit 2 (Day 15)	160 (77.3)	30 (14.5)	17 (8.2)	46	33 (71.7)	8 (17.4)	5 (10.9)	0.172	148	117 (79.1)	20 (13.5)	11 (7.4)	0.998
Visit 3 (Day 30)	162 (79.8)	25 (12.3)	16 (7.9)		38 (82.6)	4 (8.7)	4 (8.7)			116 (78.4)	21 (14.2)	11 (7.4)	
The feeding appeared to satisfy my baby’s hunger													
Visit 2 (Day15)	175 (84.1)	26 (12.5)	7 (3.4)	46	40 (87)	2 (4.3)	4 (8.7)	0.392	148	123 (83.1)	22 (14.9)	3 (2)	**0**.**035**
Visit 3 (Day 30)	167 (82.7)	19 (9.4)	16 (7.9)		38 (82.6)	3 (6.5)	5 (10.9)			122 (82.4)	15 (10.1)	11 (7.4)	
My baby spit up with feedings													
Visit 2 (Day15)	26 (12.5)	77 (37)	105 (50.5)	46	3 (6.5)	23 (50)	20 (43.5)	0.392	149	21 (14.1)	50 (33.6)	78 (52.3)	0.947
Visit 3 (Day 30)	24 (11.8)	77 (37.9)	102 (50.2)		2 (4.3)	22 (47.8)	22 (47.8)			22 (14.8)	52 (34.9)	75 (50.3)	
Stool odor was very bad													
Visit 2 (Day15)	93 (44.9)	50 (24.2)	64 (30.9)	45	28 (62.2)	8 (17.8)	9 (20)	0.062	148	62 (41.9)	40 (27)	46 (31.1)	0.354
Visit 3 (Day 30)	91 (45.0)	36 (17.8)	75 (37.1)		23 (51.1)	6 (13.3)	16 (35.6)			65 (43.9)	30 (20.3)	53 (35.8)	
My baby’s stool consistency was just right													
Visit 2 (Day15)	102 (49.8)	59 (28.8)	44 (21.5)	45	24 (53.3)	11 (24.4)	10 (22.2)	0.801	147	70 (47.6)	46 (31.3)	31 (21.1)	0.061
Visit 3 (Day 30)	111 (54.7)	44 (21.7)	48 (23.6)		25 (55.6)	8 (17.8)	12 (26.7)			81 (55.1)	33 (22.4)	33 (22.4)	
My baby was gassy													
Visit 2 (Day15)	46 (22.1)	50 (24)	112 (53.8)	46	9 (19.6)	9 (19.6)	28 (60.9)	0.644	149	33 (22.1)	40 (26.8)	76 (51)	0.248
Visit 3 (Day 30)	38 (18.7)	44 (21.7)	121 (59.6)		7 (15.2)	13 (28.3)	26 (56.5)			31 (20.8)	30 (20.1)	88 (59.1)	
My baby’s stools were too hard													
Visit 2 (Day15)	4 (1.9)	29 (14.1)	173 (84)	45	–	1 (2.2)	44 (97.8)	–	148	4 (2.7)	26 (17.6)	118 (79.7)	0.211
Visit 3 (Day 30)	3 (1.5)	24 (11.9)	175 (86.6)		–	2 (4.4)	43 (95.6)			3 (2)	21 (14.2)	124 (83.8)	
My baby had difficulty moving bowels													
Visit 2 (Day15)	30 (14.4)	50 (24)	128 (61.5)	46	4 (8.7)	7 (15.2)	35 (76.1)	0.532	149	23 (15.4)	43 (28.9)	83 (55.7)	0.654
Visit 3 (Day 30)	25 (12.3)	42 (20.7)	136 (67)		4 (8.7)	4 (8.7)	38 (82.6)			21 (14.1)	38 (25.5)	90 (60.4)	

Values in bold indicate statistical significance (*p* < 0.05).

^a^ Data collected on a 5-point scale, but combined into Always (Always + Frequently), Sometimes, and Never (Rarely + Never).

Mcnemar Bowker test.

### Formula satisfaction

According to “Formula Satisfaction Questionnaire”, the majority of parents were satisfied with the study formula (93.2%), and reported their infants did well or very well on the formula (92.7%) with no problems encountered during formula feeding (90.2%), and wished to continue using it (92.2%). No significant change was noted between visit 2 and visit 3 in terms of formula satisfaction in formula-fed and mixed-fed groups (Table 7).

**Table 7:** Formula satisfaction questionnaire.

Formula satisfaction^a^		Overall		*n*	Exclusively formula-fed		*p*-value	*n*	Mixed-fed		*p*-value
		Visit 2 (day 15)	Visit 3 (day 30)		Visit 2 (day 15)	Visit 3 (day 30)			Visit 2 (day 15)	Visit 3 (day 30)	
Overall, how satisfied were you with the formula?	Yes	197 (93.4)	192 (93.2)	48	45 (93.8)	45 (93.8)	1.000	152	141 (92.8)	142 (93.4)	1.000
	No	14 (6.6)	14 (6.8)		3 (6.2)	3 (6.2)			11 (7.2)	10 (6.6)	
Would you want to continue using the formula?	Yes	201 (95.7)	189 (92.2)	48	46 (95.8)	45 (93.8)	1.000	150	144 (96.8)	137 (91.3)	0.065
	No	9 (4.3)	16 (7.8)		2 (4.2)	3 (6.2)			6 (4)	13 (8.7)	
How did your baby do on the formula?	Good	201 (95.3)	191 (92.7)	48	47 (97.9)	44 (91.7)	0.250	152	144 (94.7)	141 (92.8)	0.453
	Bad	10 (4.7)	15 (7.3)		1 (2.1)	4 (8.3)			8 (5.3)	11 (7.2)	
Did your baby have any problems while on the formula?	Yes	27 (12.8)	20 (9.8)	47	42 (89.4)	42 (89.4)	1.000	152	131 (86.2)	137 (90.1)	0.286
	No	184 (87.2)	185 (90.2)		5 (10.6)	5 (10.6)			21 (13.8)	15 (9.9)	
Did your baby seem to like the formula?	Yes	177 (83.9)	171 (83.0)	48	43 (89.6)	42 (87.5)	1.000	152	124 (81.6)	125 (82.2)	1.000
	No	34 (16.1)	35 (17)		5 (10.4)	6 (12.5)			28 (18.4)	27 (18.8)	
How would you describe the odor of the formula?	Good	48 (22.7)	51 (24.9)	47	10 (21.3)	10 (21.3)	1.000	152	32 (21.1)	37 (24.3)	0.302
	Bad	163 (77.3)	154 (75.1)		37 (78.7)	37 (78.7)			120 (78.9)	115 (75.7)	
How would you describe the consistency of the formula?	Fluid	193 (91.5)	184 (89.8)	48	43 (89.6)	41 (85.4)	0.500	151	138 (91.4)	138 (91.4)	1.000
	Dense	18 (8.5)	21 (10.2)		5 (10.4)	7 (14.6)			13 (8.6)	13 (8.6)	
How well did the powder mix with water?	Good	198 (93.8)	193 (93.7)	48	45 (93.8)	46 (95.8)	1.000	152	143 (94.1)	141 (92.8)	0.754
	Bad	13 (6.2)	13 (6.3)		3 (6.2)	2 (4.2)			9 (5.9)	11 (7.2)	

^a^ Data collected on a 5-point scale, but combined into Yes (Very satisfied + Satisfied + Somewhat Satisfied) and No (Very dissatisfied + Dissatisfied).

McNemar’s test.

### Adverse events

Adverse events were reported in 8 infants including vomiting in 3 of them (Table 8).

**Table 8:** Adverse events.

	Patients (226)
Adverse events, *n* (%)	
Vomiting	3 (1.3)
Rota virus infection	1 (0.4)
Cough	1 (0.4)
Urinary tract infection	1 (0.4)
Acute urticaria	1 (0.4)
Upper respiratory tract infection	1 (0.4)

## Discussion

Our findings revealed that the use of eHCF in infants with CMPA during the first 6 months of life was associated with a normal growth profile, improved WFA and WFL *z*-scores, favorable GI tolerance and a high level of parental satisfaction. There was an increase in daily frequency and amount of formula consumption over time, without a concomitant increase in the rate of spit-up and/or vomiting. At least half of the infants never experienced irritability or feeding refusal and spit-up after feeding and had normal stool consistency, while the majority of mothers considered the study formula to be satisfactory. Considering the outcomes in exclusively formula-fed and mixed-fed groups, both feeding patterns were associated with an increase in daily volume intake per each feeding, amelioration of perianal erythema, improved compliance and gastrointestinal tolerance, and high parental satisfaction with the formula. Nonetheless, the mixed-fed group seemed to be more advantageous particularly in terms of improvement in WFA and WFL *z*-scores, a decrease in frequencies of spit-up and vomiting after feeding, and a decrease in anal fissure.

Similarly, in a prospective multicenter trial in 30 infants (aged <12 months) with CMPA who received a thickened eHCF for four months, the authors reported that eHCF was tolerated by more than 90% of infants and was associated with significantly increased WFA, LFA and WFL *z*-scores during the study period (11). The authors also noted a significant increase in the percentage of infants having normal stool consistency (from 66.7% at inclusion to 90.0%) and a decrease in the rate of vomiting by 50% after 14 days of feeding along with no adverse event related to the eHCF (19). Hence, the thickened eHCF is considered to be a hypoallergenic, efficient and safe alternative in children with CMPA as associated with improvement of growth indices and absence of related adverse events (11).

The association of eHCF feeding with an improvement in growth indices was reported in other studies in infants with CMPA, and eHCF feeding was considered to enable growth normalization in line with WHO standards (19, 20, 21). Demonstrating growth in line with WHO standards for infants consuming eHCF is important given that CMPA is frequently associated with a growth deficit due to reduced bioavailability or loss of nutrients in the gastrointestinal tract, increased metabolic needs as well as the inadequate elimination diet (5, 11, 14, 15, 20, 23).

Also, in a 12-month growth and accession of tolerance study in 116 infants with CMPA, the authors reported that eHF was associated with significantly improved WFA *z*-score, while the growth improvement was more likely in infants with one symptom at diagnosis, those who had a gastrointestinal symptom, and those with an allergy to only CMP (19). This seems notable given the predominance of gastrointestinal manifestations either alone or together with other complaints and the presence of allergy to only CMP in a majority of infants in the present cohort.

In the current study, eHCF-based growth improvement was evident for WFA and WFL *z*-scores with no significant change from baseline to day 30 for LFA scores, supporting that weight gain occurs more rapidly and earlier than linear growth after nutritional interventions with alternative formulae (19, 24).

Bitter taste and poor digestive comfort including regurgitations are the two problems frequently encountered with use of eHF (11, 25–28). In the current study, >80% of parents reported that infants liked the formula, and at least half of infants never experienced feeding refusal and spit-up after feeding, along with the increase in daily amount of formula consumption over time. Hence, our findings support the previously reported high parent satisfaction rates with eHCF, particularly in terms of their child’s acceptance of the formula’s taste (11).

Moreover, in addition to enabling normal stool consistency with no bloating or flatulence in at least half of infants, eHCF also revealed feelings of satiety, normal feeding time and soft and formed stools and no difficulty in defecating in the majority of our study population along with a significant decrease in the rate of anal fissure and perianal erythema. Notably, the mixed-fed group had certain advantages over the exclusively formula-fed group in terms of improvement in WFA and WFL *z*-scores, decrease in the frequency of spit-up and/or vomiting after feeding and amelioration of anal fissure over time, despite the higher likelihood of having multiple food allergies and gastrointestinal + cutaneous + respiratory manifestations at the initial presentation.

Data from small case series indicated up to 10% of children with CMPA could react adversely to eHCF (29, 30). Our findings indicate adverse events in 8(3.5%) of infants with vomiting as the leading event (3 of 8 patients), while parents reported no additional problems during feeding in 87.2% and 90.2% of infants on day 15 and day 30 of eHCF intervention, respectively. In a study among infants with CMPA receiving eHCF, 33 AEs were reported in 24 patients including respiratory infections (48.5%) and gastroenteritis (30.0%), while none were related to the tested formula nor led to feeding discontinuation of the tested formula (19). Likewise, in a study with 220 children with CMPA who received eHCF or eHCF plus LGG, authors reported no adverse reactions to any of the study formulas (31).

In a systematic review of 15 RCTs in 1,285 children with CMPA on the use of a formula containing eHF (whey and/or casein) or any other formula for CMPA management, the authors concluded that eHF products appear to be well-tolerated by most children with CMPA (32). However, they also noted that there are numerous methodological issues preventing to reach a conclusion regarding the benefit of one formula over another formula, such as the differences in outcome measures and their definitions, lack of pre-specified protocols and/or trial registration, and poor reporting of adverse events (32). Hence, the authors emphasized the need for standardized treatment protocols and a standardized set of outcomes to be measured and reported in clinical trials addressing the utility of specialized milk formula for the management of CMA (32).

The major strength of this observational study seems to be the nationwide inclusion of infants with CMPA from 35 pediatric gastroenterology and allergy centers across Turkey which enables our findings to be likely to be generalizable with a representative sample of the overall CMPA population. In addition, the current study provides evidence on the management of infants with CMPA via a comprehensive assessment including not only the gastrointestinal tolerance and safety of the eHCF but also the infant growth, infant feeding and stool patterns and the parental satisfaction with the formula, both in exclusively formula-fed and mixed-fed infants. However, certain limitations to this study should be considered. First, single-arm design with no control group is an important limitation. Second, the short duration of follow up is another limitation. Third, while not included within the scope of the current study, data on changes in individual presenting symptoms over the course of feeding with eHCF might extend the knowledge achieved in the current study.

## Conclusion

In conclusion, our findings indicate that eHCF was well-accepted and tolerated by an intended-use population of infants with CMPA during the first 6 months of life and enabled adequate volume consumption and improved growth indices even within 30 days of utilization alongside favorable gastrointestinal tolerance and a high level of parental satisfaction. Future longer-term and larger-scale studies are warranted to assess the utility of eHCF in infants with CMPA in terms of growth indices, development of tolerance and amelioration of gastrointestinal and allergy symptoms.

## Data Availability

The original contributions presented in the study are included in the article, further inquiries can be directed to the corresponding author.

## ISPAT Study Group

Ankara University School of Medicine, Ankara; Aydan Kansu (Pediatric Gastroenterology), Ceyda Tuna Kırsaclioglu (Pediatric Gastroenterology), Adana State Training and Research Hospital, Adana; Didem Gulcu Taskin (Pediatric Gastroenterology), Afyonkarahisar Health Sciences University Faculty of Medicine, Afyonkarahisar; Aysegul Bukulmez (Pediatric Gastroenterology), Lutfi Molon (Pediatrics), Akdeniz Univesity Faculty of Medicine, Antalya; Reha Artan (Pediatric Gastroenterology), Aysen Bingol (Pediatric Immunology and Allergy), Arzu Aras (Pediatric Gastroenterology), Dilara Kocacik Uygun (Pediatric Immunology and Allergy), Aygen Yilmaz (Pediatric Gastroenterology), Ankara Diskapi Children’s Hospital, Ankara; Arzu Meltem Demir (Pediatric Gastroenterology), Burcu Berberoglu Ates (Pediatric Gastroenterology), Ankara Sami Ulus Maternity and Child Health and Diseases Training and Research Hospital, Ankara; Ilknur Bostanci (Pediatric Immunology and Allergy), Aysegul Ertugrul (Pediatric Immunology and Allergy), Ezgi Ulusoy Severcan (Pediatric Immunology and Allergy), Ankara Training and Research Hospital, Ankara; Emine Vezir (Pediatric Gastroenterology), Hayriye Hizarcioglu Gulsen (Pediatric Gastroenterology), Ankara Yildirim Beyazit University Faculty of Medicine, Ankara; Samil Hizli (Pediatric Gastroenterology), Balikesir University Faculty of Medicine, Balikesir; Sezin Akman (Pediatric Gastroenterology), Gulce Baranli (Pediatric Gastroenterology), Baskent University Hospital, Konya; Bahar Gokturk (Pediatric Immunology and Allergy), Erzurum Regional Training and Research Hospital, Erzurum; Asuman Nur Karhan (Pediatric Gastroenterology), Firat University Faculty of Medicine, Elazig; Yasar Dogan (Pediatric Gastroenterology), Hatice Altug Demirol (Pediatric Gastroenterology), Gazi University Faculty of Medicine, Ankara; Buket Dalgic (Pediatric Gastroenterology), Hakan Ozturk (Pediatric Gastroenterology), Gaziantep University Faculty of Medicine, Gaziantep; Ahmet Basturk (Pediatric Gastroenterology), Hacettepe University Faculty of Medicine, Ankara, Hasan Ozen (Pediatric Gastroenterology), Bulent Enis Sekerel (Pediatric Allergy), Betul Buyuktiryaki (Pediatric Allergy), Ersin Gümüş (Pediatric Gastroenterology), Health Sciences University Bursa Yuksek Ihtisas Training and Research Hospital, Bursa; Kaan Demiroren (Pediatric Gastroenterology), Havva Hasret Cagan (Pediatric Gastroenterology), Inonu University Faculty of Medicine, Malatya Fatma Ilknur Varol (Pediatric Gastroenterology), Senay Kenc (Pediatric Gastroenterology), Istanbul Atakent Acibadem Hospital, Istanbul; Vildan Ertekin (Pediatric Gastroenterology), Istanbul Bakirkoy Sadi Konuk Training and Research Hospital, Istanbul; Hasret Ayyildiz (Pediatric Gastroenterology), Lida Bülbül (Pediatric Gastroenterology), Istanbul Esenler Maternity and Child Health and Diseases Hospital; Istanbul; Mahmut Caner Us, Erkan Akkuş, Istanbul Goztepe Training and Research Hospital, Istanbul; Mustafa Arga (Pediatric Allergy), Ozlem Cavkaytar (Pediatric Allergy), Istanbul Okmeydani Training and Research Hospital, Istanbul; Birol Ozturk (Pediatric Gastroenterology), Istanbul Sisli Hamidiye Etfal Training and Research Hospital, Istanbul; Aysenur Kaya (Pediatric Immunology and Allergy), Nafiye Urganci (Pediatric Gastroenterology), A. Merve Usta (Pediatric Gastroenterology), Lutfiye Sahin Keskin (Pediatric Gastroenterology), Gokce Kuru (Pediatric Gastroenterology), Istanbul Umraniye Training and Research Hospital, Istanbul; Nelgin Gerenli (Pediatric Gastroenterology), Esra Polat (Pediatric Gastroenterology), Istanbul University Cerrahpasa Faculty of Medicine, Istanbul; Tufan Kutlu (Pediatric Gastroenterology), Hasan Karakas (Pediatric Gastroenterology), Kanuni Sultan Suleyman Training and Research Hospital, Istanbul; Gunsel Kutluk (Pediatric Gastroenterology), Karadeniz Teknik University Faculty of Medicine, Trabzon Murat Cakir (Pediatric Gastroenterology), Elif Sag (Pediatric Gastroenterology), Kirikkale University Faculty of Medicine, Kirikkale; Hacer Fulya Gulerman (Pediatric Gastroenterology), Zeynep Arslan (Pediatric Gastroenterology), Dilek Azkur (Pediatric Gastroenterology), Kocaeli University Faculty of Medicine, Kocaeli; Aysen Uncuoglu Aydogan (Pediatric Gastroenterology), Nihal Uyar Aksu (Pediatric Gastroenterology), Konya Necmettin Erbakan University Meram Faculty of Medicine, Konya; Sukru Nail Guner (Pediatric Allergy), Hasan Kapakli (Pediatric Allergy), Konya Selcuk University Faculty of Medicine, Konya; Halil Haldun Emiroglu (Pediatric Gastroenterology), Mehmet Akif Agir (Pediatric Gastroenterology), Meltem Gumus (Pediatric Gastroenterology), Alaaddin Yorulmaz (Pediatric Gastroenterology), Pamukkale University Faculty of Medicine, Denizli; Halil Kocamaz (Pediatric Gastroenterology), Ebru Arik Yilmaz (Pediatric Immunology and Allergy), Eda Didem Kayakiran (Pediatric Gastroenterology), Samsun Training and Research Hospital, Samsun; Mahir Igde (Pediatric Immunology and Allergy), Ozlem Yuce Kirmemiş (Pediatric Immunology and Allergy), Trabzon Kanuni Training and Research Hospital, Trabzon Aysegul Akan (Pediatric Immunology and Allergy), Zonguldak Bulent Ecevit University Faculty of Medicine, Zonguldak; Mutlu Yuksek (Pediatric Immunology and Allergy), Aysugul Alptekin Sarioglu, Simge Erdogan, Abbott Nutrition, Abbott Laboratories.
